# Supplementary material for: Caries risk assessment using different Cariogram models. A comparative study about concordance in different populations—Adults and children
Source: PLoS One. 2022 Jun 24;17(6):e0264945. doi: 10.1371/journal.pone.0264945 (PMC9231745; doi:10.1371/journal.pone.0264945)
Supplement: S1 Appendix — (DOC) [file pone.0264945.s002.doc]

| Patient identification number | | | | |
| --- | --- | --- | --- | --- |
|  |  |  |  |  |

|  |  |  |  |
| --- | --- | --- | --- |

Data___/____/____

ABITUDINI/COMPORTAMENTO IN MATERIA DI SALUTE ORALE

| Examiner: |
| --- |

Cognome:_____________________________ Nome__________________________ Data di Nascita:______________

Field:__________________________________ Camp:_________________________ Genere: 🞏 🞏

M F

1. Che lavoro fa sua madre? □ □

SI NO

1. Che lavoro fa suo padre? □ □

SI NO

1. Durata in anni, del percorso di studio di sua madre? _______________________
2. Durata in anni, del percorso di studio di suo padre? ________________________
3. Quante persone vivono nella tua casa? ? □ □ □ □ □ □ ____

1 2 3 4 5 6

1. Quante volte mangi al giorno? □ □ □ □
2. 2 3 4

7. Spazzoli I denti dopo ogni pasto? □ □ □

SI NO QUALCHE VOLTA

8. Mangi dolci snack tra i pasti principali ?Do you eat sweets or sweet snacks during your meals? □ □ □

SI NO QUALCHE VOLTA

9. Mangia dolci o spuntini dolci tra i pasti? □ □ Se SI quanto spesso? □ □ □ □

SI NO 1 2 3 4

1. Bevi bevande analcoliche durante i tuoi pasti Do you drink soft drinks during your meals? □ □ □

SI NO QUALCHE VOLTA

1. Bevi soft-drinks tra i pasti principali? □ □ Se SI quanto spesso? □ □ □ □

SI NO         1 2 3 4

1. Usi spazzolino e dentifricio? □ □

SI NO

1. Quante volte al giorno, spazzoli i tuoi denti? □ □ □ □

1 2 3 4

1. Mai avuto mal di denti? □ □

SI NO

1. Mai andato dal dentista? □ □

SI NO

1. I tuoi genitori usano spazzolino e dentifricio? □ □

SI NO

1. I tuoi genitori vanno regolarmente dal dentista? □ □

SI NO
