# Supplementary material for: Caries risk assessment using different Cariogram models. A comparative study about concordance in different populations—Adults and children
Source: PLoS One. 2022 Jun 24;17(6):e0264945. doi: 10.1371/journal.pone.0264945 (PMC9231745; doi:10.1371/journal.pone.0264945)
Supplement: S2 Appendix — (DOC) [file pone.0264945.s003.doc]

| Patient identification number | | | | |
| --- | --- | --- | --- | --- |
|  |  |  |  |  |

|  |  |  |  |
| --- | --- | --- | --- |

Date___/____/____

HABITS/BEHAVIOUR REGARDING ORAL HEALTH

| Examiner: |
| --- |

Surname:_____________________________ Name__________________________ Date of birth:______________

Field:__________________________________ Camp:_________________________ Sex: 🞏 🞏

M F

1. Does your mother work? □ □

YES NO

1. Does your father work? □ □

YES NO

1. How many years did your mother study? _______________________
2. How many years did your father study? ________________________
3. How many people live in your house? ? □ □ □ □ □ □ ____

1 2 3 4 5 6

1. How many times do you eat in a day? □ □ □ □

1 2 3 4

1. Do you brush your teeth every time you eat? □ □ □

YES NO SOMETIMES

1. Do you eat sweets or sweet snacks during your meals? □ □ □

YES NO SOMETIMES

1. Do you eat sweets or sweet snacks between meals? □ □ If yes how often? □ □ □ □

YES NO 1 2 3 4

1. Do you drink soft drinks during your meals? □ □ □

YES NO SOMETIMES

1. Do you drink soft drinks between meals? □ □ If yes how often? □ □ □ □

YES NO         1 2 3 4

1. Do you use a toothbrush and toothpaste? □ □

YES NO

1. How many times a day do you brush your teeth? □ □ □ □

1 2 3 4

1. Have you ever had a toothache? □ □

YES NO

1. Do you ever go to the dentist’s? □ □

YES NO

1. Do your parents brush their teeth? □ □

YES NO

1. Do your parents go to the dentist’s? □ □

YES NO
