# Supplementary material for: Caries risk assessment using different Cariogram models. A comparative study about concordance in different populations—Adults and children
Source: PLoS One. 2022 Jun 24;17(6):e0264945. doi: 10.1371/journal.pone.0264945 (PMC9231745; doi:10.1371/journal.pone.0264945)
Supplement: Attachment — Submitted filename: PONE-D-21-19761_reviewer.pdf [file pone.0264945.s005.pdf]

PLOS ONE

Caries risk assessment using different Cariogram models. A comparative study about concordance in different populations - adults and children.

--Manuscript Draft--

Manuscript Number:	PONE-D-21-19761
Article Type:	Research Article
Full Title:	Caries risk assessment using different Cariogram models. A comparative study about concordance in different populations - adults and children.
Short Title:	Comparison of different Cariogram models in different populations
Corresponding Author:	Guglielmo Campus<br>University of Bern: Universitat Bern<br>Bern, SWITZERLAND
Keywords:	caries risk assessment; Cariogram; Reliability; Concordance
Abstract:	Background: This methodological survey aimed to verify whether there is concordance among several Cariogram different risk assessements at different cut-off values, comparing both children and adult popualtions; and how each risk/protective factor weight on the overall caries risk profile.<br>Methods: Three groups' data (two in children and one in adults) were obtained from previous studies, while a fourth, in young adults, was ad hoc enrolled. Different caries risk levels were assessed: a) three risk categories with two different cut-offs as: "low risk"=61-100% or 81-100% chance to avoid caries, "moderate risk"=41-60% or 21-80% and "high risk"=0-40% or 0-20%, named model 1 and 2; b) four risk categories with two different cut-offs as: "low risk"=61-100% or 76-100%, "moderate/low risk"=41-60% or 51-75%; "moderate/high risk"=21-40% or 26-50% and "high risk"=0-20% or 0-25%, model 3 and 4; c) five risk categories as: "very low risk"=81-100%; "low risk"=61-80% "moderate risk"=41-60%; "high risk"=21-40% and "very high risk"=0-20%, model 5. Concordance of the different Cariogram risk categories among the four groups was calculated using Cohen's kappa. The weight of the association between all Cariogram models toward the Cariogram risk variables was evaluated by ordinal logistic regression models.<br>Results: Considering Cariogram models 1 and 2, Cohen's Kappa values ranged from 0.40 (SE=0.07) for the young adult group to 0.71 (SE=0.05) for the adult one. Cohen's Kappa values ranged from 0.14 (SE=0.03 p<0.01) for the adult group to 0.62 (SE=0.02) for the two groups of children in models 3 and 4. Statistically significant associations were found for all Cariogram risk variables excepting Fluoride program – in models 4 and 5 and overall risk on children's samples. Caries experience showed a quite variable weight in the different models in both adult groups. In the regression analyses, adult groups' convergence was not always achievable since variations in associations between caries risk and different risk variables were narrower compared to other samples.<br>Conclusion: Significant differences in caries risk stratification using different cut-offs stands out from data analysis; consequently, risk assessments need to be carefully considered due to the risk of misleadingly choosing preventive and research actions.
Order of Authors:	Maria Grazia Cagetti
	Giuliana Bontà
	Juan Sebastian Lara
	Guglielmo Campus
Additional Information:	
Question	Response
**Financial Disclosure**<br><br>Enter a financial disclosure statement that describes the sources of funding for the	The authors received no specific funding for this work.

Powered by Editorial Manager® and ProduXion Manager® from Aries Systems Corporation

work included in this submission. Review the submission guidelines for detailed requirements. View published research articles from *PLOS ONE* for specific examples.<br><br>This statement is required for submission and **will appear in the published article** if the submission is accepted. Please make sure it is accurate.<br><br>**Unfunded studies**<br>Enter: *The author(s) received no specific funding for this work.*<br><br>**Funded studies**<br>Enter a statement with the following details:<br>• Initials of the authors who received each award<br>• Grant numbers awarded to each author<br>• The full name of each funder<br>• URL of each funder website<br>• Did the sponsors or funders play any role in the study design, data collection and analysis, decision to publish, or preparation of the manuscript?<br>• **NO** - Include this sentence at the end of your statement: *The funders had no role in study design, data collection and analysis, decision to publish, or preparation of the manuscript.*<br>• **YES** - Specify the role(s) played.<br><br>* typeset	
**Competing Interests**<br><br>Use the instructions below to enter a competing interest statement for this submission. On behalf of all authors, disclose any competing interests that could be perceived to bias this work—acknowledging all financial support and any other relevant financial or non-financial competing interests.<br><br>This statement is required for submission and **will appear in the published article** if the submission is accepted. Please make sure it is accurate and that any funding sources listed in your Funding Information	The authors have declared that no competing interests exist.

Powered by Editorial Manager® and ProduXion Manager® from Aries Systems Corporation

later in the submission form are also declared in your Financial Disclosure statement.<br><br>View published research articles from *PLOS ONE* for specific examples.<br><br>**NO authors have competing interests**<br><br>Enter: *The authors have declared that no competing interests exist.*<br><br>**Authors with competing interests**<br><br>Enter competing interest details beginning with this statement:<br><br>*I have read the journal's policy and the authors of this manuscript have the following competing interests: [insert competing interests here]*<br><br>* typeset	
**Ethics Statement**<br><br>Enter an ethics statement for this submission. This statement is required if the study involved:<br><br>• Human participants<br>• Human specimens or tissue<br>• Vertebrate animals or cephalopods<br>• Vertebrate embryos or tissues<br>• Field research<br><br>Write "N/A" if the submission does not require an ethics statement.<br><br>General guidance is provided below. Consult the submission guidelines for detailed instructions. **Make sure that all information entered here is included in the Methods section of the manuscript.**	Name of the institutional review board or ethics committee: "Comitato Etico Milano Area 1" email: comitato.etico@asst-fbf-sacco.it P/03/06/2020<br>Approval n. 2019/ST/167.

Powered by Editorial Manager® and ProduXion Manager® from Aries Systems Corporation

**Format for specific study types**<br><br>**Human Subject Research (involving human participants and/or tissue)**<br>• Give the name of the institutional review board or ethics committee that approved the study<br>• Include the approval number and/or a statement indicating approval of this research<br>• Indicate the form of consent obtained (written/oral) or the reason that consent was not obtained (e.g. the data were analyzed anonymously)<br><br>**Animal Research (involving vertebrate animals, embryos or tissues)**<br>• Provide the name of the Institutional Animal Care and Use Committee (IACUC) or other relevant ethics board that reviewed the study protocol, and indicate whether they approved this research or granted a formal waiver of ethical approval<br>• Include an approval number if one was obtained<br>• If the study involved *non-human primates*, add *additional details* about animal welfare and steps taken to ameliorate suffering<br>• If anesthesia, euthanasia, or any kind of animal sacrifice is part of the study, include briefly which substances and/or methods were applied<br><br>**Field Research**<br><br>Include the following details if this study involves the collection of plant, animal, or other materials from a natural setting:<br>• Field permit number<br>• Name of the institution or relevant body that granted permission	
**Data Availability**<br><br>Authors are required to make all data underlying the findings described fully available, without restriction, and from the time of publication. PLOS allows rare exceptions to address legal and ethical concerns. See the PLOS Data Policy and FAQ for detailed information.	Yes - all data are fully available without restriction

Powered by Editorial Manager® and ProduXion Manager® from Aries Systems Corporation

A Data Availability Statement describing where the data can be found is required at submission. Your answers to this question constitute the Data Availability Statement and **will be published in the article**, if accepted.<br><br>**Important:** Stating 'data available on request from the author' is not sufficient. If your data are only available upon request, select 'No' for the first question and explain your exceptional situation in the text box.<br><br>Do the authors confirm that all data underlying the findings described in their manuscript are fully available without restriction?	
**Describe where the data may be found in full sentences. If you are copying our sample text, replace any instances of XXX with the appropriate details.**<br><br>• If the data are **held or will be held in a public repository**, include URLs, accession numbers or DOIs. If this information will only be available after acceptance, indicate this by ticking the box below. For example: *All XXX files are available from the XXX database (accession number(s) XXX, XXX.*).<br>• If the data are all contained **within the manuscript and/or Supporting Information files**, enter the following: *All relevant data are within the manuscript and its Supporting Information files.*<br>• If neither of these applies but you are able to provide **details of access elsewhere**, with or without limitations, please do so. For example:<br><br>*Data cannot be shared publicly because of [XXX]. Data are available from the XXX Institutional Data Access / Ethics Committee (contact via XXX) for researchers who meet the criteria for access to confidential data.*<br><br>*The data underlying the results presented in the study are available from (include the name of the third party*	All relevant data are within the manuscript and its Supporting Information files.

and contact information or URL).<br>• This text is appropriate if the data are owned by a third party and authors do not have permission to share the data.<br><br>* typeset	
Additional data availability information:	

Manuscript Click here to access/download;Manuscript;Manuscript.docx

Caries risk assessment using different Cariogram models. A comparative study about concordance in different populations - adults and children.

Maria Grazia Cagetti[1], Giuliana Bontà[1], Juan Sebastian Lara[2], Guglielmo Campus*[3,3,5]

[1]Department of Biomedical, Surgical and Dental Science, University of Milan, Via Beldiletto 1, 20142 Milan, Italy

[2]Department of Cariology, Operative Dentistry and Dental Public Health. Indiana University School of Dentistry. Indianapolis, IN, USA

[3]Department of Restorative, Preventive and Pediatric Dentistry, University of Bern, 3012 Bern, Switzerland

[4]Department of Surgery, Microsurgery and Medicine Sciences, School of Dentistry, University of Sassari, Viale San Pietro 3/c, 07100 Sassari, Italy

[5]School of Dentistry, Sechenov University, 119991 Moscow, Russia

*Corresponding author:
Prof Guglielmo Campus
Department of Restorative, Preventive and Pediatric Dentistry,
University of Bern,
Freiburgstrasse 7,
CH 3010 Bern, Switzerland
guglielmo.campus@zmk.unibe.ch

Abstract:

Background: This methodological survey aimed to verify whether there is concordance among several Cariogram different risk assessements at different cut-off values, comparing both children and adult popualtions; and how each risk/protective factor weight on the overall caries risk profile.

Methods: Three groups' data (two in children and one in adults) were obtained from previous studies, while a fourth, in young adults, was *ad hoc* enrolled. Different caries risk levels were assessed: a) three risk categories with two different cut-offs as: "low risk"=61-100% or 81-100% chance to avoid caries, "moderate risk"=41-60% or 21-80% and "high risk"=0-40% or 0-20%, named model 1 and 2; b) four risk categories with two different cut-offs as: "low risk"=61-100% or 76-100%, "moderate/low risk"=41-60% or 51-75%; "moderate/high risk"=21-40% or 26-50% and "high risk"=0-20% or 0-25%, model 3 and 4; c) five risk categories as: "very low risk"=81-100%; "low risk"=61-80% "moderate risk"=41-60%; "high risk"=21-40% and "very high risk"=0-20%, model 5. Concordance of the different Cariogram risk categories among the four groups was calculated using Cohen's kappa. The weight of the association between all Cariogram models toward the Cariogram risk variables was evaluated by ordinal logistic regression models.

Results: Considering Cariogram models 1 and 2, Cohen's Kappa values ranged from 0.40 (SE=0.07) for the young adult group to 0.71 (SE=0.05) for the adult one. Cohen's Kappa values ranged from 0.14 (SE=0.03 p<0.01) for the adult group to 0.62 (SE=0.02) for the two groups of children in models 3 and 4. Statistically significant associations were found for all Cariogram risk variables excepting Fluoride program – in models 4

and 5 and overall risk on children's samples. Caries experience showed a quite variable weight in the different models in both adult groups. In the regression analyses, adult groups' convergence was not always achievable since variations in associations between caries risk and different risk variables were narrower compared to other samples.

Conclusion: Significant differences in caries risk stratification using different cut-offs stands out from data analysis; consequently, risk assessments need to be carefully considered due to the risk of misleadingly choosing preventive and research actions.

Key words: caries risk assessment, Cariogram, Reliability, Concordance

Background

Assessing and classifying individual caries risk is an essential step in the caries diagnosis and decision-making process to achieve the best non-invasive and conservative restorative management options as well as establishing recall intervals, controlling and reducing the incidence of the disease [1, 2].

Caries risk assessment (CRA) should be an integral part of the treatment regimens, in order to plan an individualized caries prevention strategy. However, routinely the patient caries risk profile is not always determined and, when performed, the risk level is not always linked to the adopted preventive and intervention strategies [3].

From the 2000s onwards, a variety of standardized tools were developed and tested in different populations [4,5]. All multifactorial risk tools include a different combination of risk and protective factors, attributing to each variable a different "weight" in the caries risk calculation [6]. Despite this, the scientific evidence on CRA tools' validity remains limited [4,5,7]. Overall, Cariogram is the only multidimensional tool verified in children and adults in few good quality studies, and its accuracy level was found to be limited in different age groups [4]. Moreover, Cariogram has been applied in different ways: considering seven to ten risk variables, considering three to five risk categories and finally, considering different cut-offs to define each risk category [8-10]. While some studies have attested the Cariogram effectiveness when used with a reduced number of factors, mostly excluding salivary factors [11-15], no study has investigated whether the use of different risk categories considering different ranges of percentages to avoid caries, produces concurrent results. In view of the foregoing, the present methodological study aimed primary, to verify if there is

concordance among the Cariogram caries risk categories at different cut-off points in children and adults; and secondly, how much each risk/protective variables weight for the different overall caries risk profile results in different age groups.

Material and Methods

Samples

Four sample groups were considered in the comparisons. Two were obtained from previous studies in children [11, 14], one in adults [15], and the fourth one, which included young adults, was *ad hoc* enrolled.

In children's groups, a reduced Cariogram model (excluding saliva parameters: buffer capacity and saliva secretion rate) was build-up to assess the forecast probabilities of new caries. Two years later, the same sample was re-examined and the caries risk re-calculated. The children samples consisted of 957 subjects aged 7–9 years (C_{young}) and 862 children aged 9–11 years (C_{old}).

The adult sample (A_{old}) included 480 subjects aged 30-45 years. Caries risk was calculated through the full Cariogram model. For the last sample of young adults (A_{young}), undergraduate second and third-year dental students, School of Dentistry, University of Milan, Italy, were invited to participate. This convenience young adult sample consisted of 86 subjects (mean age 23.37 ± 3.11 years).

Data collection

Data for samples C_{young}, C_{old} and A_{old}, were extracted from the previously published studies. Data collection for sample A_{young} followed the same procedures in the adult population study [15], which included: a self-administered questionnaire collecting

data on age, oral hygiene habits (frequency of toothbrushing), dietary habits (daily intake of sugary foods and drinks), fluoride exposure in addition to toothpaste and other remineralizing and antibacterial compounds, frequency of dental checkups, presence of systemic diseases, and use of medications. Additionally, participants received clinical examinations by two examiners (GB and MGC) to determine caries prevalence at dentinal level, caries experience (Decayed, Missing and Filled Teeth index) [16] and plaque index [11]. Finally, saliva chair-side tests were performed under standardized conditions to evaluate the salivary *mutans* Streptococci and *Lactobacillus spp.* counts, and buffer capacity.

Risk assessment

Caries risk profiles were calculated using Cariogram for all subjects in the different groups. Cariogram risk for samples C_{young} and C_{old} was calculated including seven risk/protective variables: caries experience, related diseases, diet content, diet frequency, plaque amount, *mutans* Streptococci count and fluoride program. For samples A_{young} and A_{old}, in addition to the previous factors, saliva secretion rate and buffer capacity were also included.

The caries risk level was assessed with five different models (Table 1): models 1 and 2 - following three risk categories with two different cut-off values (% chance of avoid caries) as: “low risk”=61-100% or 81-100% , “moderate risk”=41-60% or 21-80% and “high risk”=0-40% or 0-20%, respectively; models 3 and 4 - following four risk categories with two different cut-off values (% chance to avoid caries): “low risk”=61-100% or 76-100%, “moderate/low risk”=41-60% or 51-75%; “moderate/high risk”=21-

40% or 26-50% and “high risk”=0-20% or 0-25%, respectively; and finally, model 5 - in five categories as follow: “very low risk”=81-100%; “low risk”=61-80% “moderate risk”=41-60%; “high risk”=21-40% and “very high risk”=0-20% chance to avoid caries, respectively.

Statistical analysis

Cohen´s Kappa values were calculated to assess the agreement of the Cariogram risk categories among the 4 groups. Kappa statistics were tested using z test at a significance level of α = 0.01. Ordinal logistic regression models were built to evaluate the weight of the associations, via Odds Ratio and $_{95\%}$Confidence Intervals, of the variables inserted on each Cariogram model toward Cariogram categorizations.

Results

Table 2 shows the demographic characteristics and caries figures of all groups. The mean caries experience (DMFT/dmft) ranged from 2.37±4.05 in the C_{young} (including both primary and permanent dentitions) to 3.25±4.15 in the A_{old}, with a D/d component ranging from 1.38±2.62 to 0.24±0.72, respectively.

Comparisons among the sample groups and the different risk categories and cut-off points are displayed in Table 3. Caries risk profiles were statistically significant different for all age groups (p values ranging from <0.01 to 0.02) when considering three (low, moderate and high) and four (low, moderate/low, moderate/high and high) risk categories. Particularly, Cohen´s Kappa values ranged from 0.71 (SE=0.05) to 0.40 (SE=0.07) in the A_{old} and the A_{young} groups, respectively when considering three

Cariogram risk categories. Children groups showed intermediate values. On the other hand, when four risk categories were considered, Cohen´s Kappa values ranged from 0.62 (SE=0.02) to 0.14 (SE=0.03 p<0.01) in both children groups and in the A_{old} group, respectively. Comparisons for the last risk model (including 5 categories: very low, low, moderate, high, very high) were not performed since only one set of cut-off values was used.

A multinomial regression analysis was run, using caries risk categories as dependent variables, to evaluate the weight of the association (OR) between the overall risk measured with the different models and the risk/protective variables considered in each model (Table 4). In the C_{young} and C_{old} groups all considered risk variables were significantly associated to the caries risk profiles, excepting Fluoride Program in models 4 and 5. However, each OR values ranged widely among models. While the weight of caries experience, *mutans* Streptococci and fluoride program remained quite stable in different models, diet content, diet frequency and plaque amount showed the widest variability in the weight of the associations. In C_{young}, diet content ranged from OR 2.48 $_{95\%}$CI 1.30 / 4.74 to OR 22.09 $_{95\%}$CI 6.23 / 78.34 in model 5 and 2, respectively; and plaque amount from OR 2.24 $_{95\%}$CI 1.53 / 3.28 to OR 299.53 $_{95\%}$CI 106.26 / 844.38 in model 1 and 2, respectively. Diet frequency for C_{old} exhibited the highest value in model 1 (OR 16.06 $_{95\%}$CI 1.94 / 13.15) and the lowest in model 4 (OR 4.54 $_{95\%}$CI 1.90 / 10.86), while plaque amount showed the highest value in model 2 (OR 405.56 $_{95\%}$CI 143.68 / 1144.80) and the lowest in model 1 (OR 1.55 $_{95\%}$CI 1.01 / 2.36). In relation to A_{young} group, caries experience showed a quite variable weight in the different models, reaching its highest value in model 3 (OR 32.49 $_{95\%}$CI 4.00 /

263.85), and the lowest in model 4 (OR 2.66 $_{95\%}$CI 1.56 / 4.52). All other risk variables resulted more strongly associated with risk profiles measured using model 1, with *mutans* Streptococci counts and fluoride program showing the widest variations among models. Finally, given the low variance across risk variables and the reduced number of subjects in some risk categories in the A_{old} group for models 2 and 4, the regression analysis was only achieved for model 1, 3 and 5. In this group, variation in weight between caries risk and different risk variables were narrower less broad compared to the above described samples. Fluoride program reached the highest OR in model 1 (3.16 95%CI 1.45 / 6.91) and Buffer capacity in model 3 and 5 (OR 7.28 95%CI 0.90 / 34.62, in both models).

Discussion

This study was designed to evaluate the concordance among caries risk profiles evaluated with Cariogram at different risk categories and different cut-off values. The study used previous published data (two samples of schoolchildren and one of adults) and original data from a fourth group of young adults. The significant difference in caries risk stratification using different cut-off points stands out as the main outcome of the present study´s data analysis.

Cariogram demonstrates the caries risk graphically and provides a percentage value illustrating the probability an individual has to avoid the development of lesions in the near future. Nevertheless, the weights attributed to the risk factors considered in the Cariogram did not appear to explain caries experience or caries activity in all groups [20].

From a public health perspective, when caries risk is assessed in population samples, it is useful to allocate each subject into a risk category, facilitating the implementation of large-scale preventive and management interventions. However, the need to allocate subjects into risk categories for community purposes, as emerges from the findings of the present methodological study, can lead to risk category allocation biases due to the number of risk categories assessed and, above all, to the cut-off values used to define each category. The allocation biases were perceived for all considered age groups. It is likely that different groups with different caries prevalence and risk variables could lead to different results. The present study findings might serve as a warning to public health dentists, epidemiologists and researchers on the adoption of Cariogram models with different cut-offs may lead to bias and an incorrect interpretation of the subject's real risk. As suggested in the original Cariogram manual [21,22] low caries risk is only determined when the "Chance to avoid cavities" is equal to 75% or higher, while a high caries risk is plausible when the 'Chance to avoid cavities' is equal to 25% or lower. . No suggestion is given for intermediate values. Nevertheless, some of the original Cariogram authors suggested, in one of the earliest papers published on Cariogram, the use of five risk categories (model 5 in the present paper) [22].

From a community or research perspective, an erroneus risk categorization could lead to unnecessary or inadequate preventive interventions [4]. This bias is confirmed by the analysis of the weight of the association between the risk/protective variables and the overall caries risk evaluated through the different five Cariogram models. The background risk factors (*i.e.* Related disease, Diet Contents, Diet Frequency, Plaque

Amounts, *mutans* Streptococci, Fluoride Program, Saliva Secretion, Buffer Capacity) had a very different association with the caries risk measured with each model. Hence, the association between the caries risk and the background variables might be miscalculated and misunderstood. “Plaque amount” is a perfect example of this; plaque amount assumes within the same sample an extremely high weight in some models and a reduced one in others. Also, the role of fluoride program is emblematic; it is not even associated to caries risk in some models (both in children and adults), while strongly associated in some of the adult models, and becomes weaker in others. On the one hand, the Cariogram assessment at the individual level, allows to graphically evaluate which factors are the haviest contributors to an individual caries risk, allowing to intervene on them. On the other hand and as previously reported, the graphical advantages of Cariogram are practically unattainableat a community level, leading to non-effective interventions decisions dfrom a clinical and/or cost / benefit point of view [23].

The outcomes of this study might allow some considerations: The caries risk classification is modified when Cariogram cut-offs varies. The interpretadtion of caries risk profiles needs to be carefully considered in dental public health given the risk of misleading the choice of both preventive and management actions, as well as the wrong selection of subjects in specific caries risk categories for research purposes.

Conflicts of interest

The Authors certify that there is no conflict of interest with any financial organization regarding the material discussed in the manuscript.

Abbreviations

dmft: Decayed, missed, filled teeth (primary dentition); DMFT: Decayed, missed, filled teeth (permanent dentition); OR: Odds ratio; 95%CI: 95% Confidence interval;

Acknowledgements

The authors want to thank the dental students of the University of Milan that participated in the study.

Authors' contributions

MGC: designed the survey and drafted the manuscript; GB: participated in the the survey'sdata collection; JSL realized tables and contributed to write the manuscript; GC: designed the survey, performed the data analysis, checked data analysis and drafted the manuscript. All authors read and approved the final version of the manuscript.

Funding

No funding was obtained for this study.

Availability of data and materials

All generated and analyzed data during this study are included in this published article and/or in additional files. The data were available as Additional file 1: Raw data.

Ethics approval and consent to participate

For the part related to the new data collection the study protocol was submitted and approved by Comitato Etico Milano Area 1 (n=2019/ST/167).

Consent for publication

Not applicable.

Competing interests

The authors declare that they have no competing interests. There are no financial completing interests as we have not received any grants. The authors alone are responsible for the content and writing of the paper.

References

1. Schwendicke F, Splieth CH, Bottenberg P, Breschi L, Campus G, Doméjean S, Ekstrand K, Giacaman RA, Haak R, Hannig M, Hickel R, Juric H, Lussi A, Machiulskiene V, Manton D, Jablonski-Momeni A, Opdam N, Paris S, Santamaria R, Tassery H, Zandona A, Zero D, Zimmer S, Banerjee A. How to intervene in the caries process in adults: proximal and secondary caries? Clin Oral Investig. 2020;24(9):3315-21. PMID: 32643090 DOI: 10.1007/s00784-020-03431-0
2. Splieth CH, Banerjee A, Bottenberg P, Breschi L, Campus G, Ekstrand KR, Giacaman RA, Haak R, Hannig M, Hickel R, Juric H, Lussi A, Machiulskiene V, Manton DJ, Jablonski-Momeni A, Opdam NJM, Paris S, Santamaría RM, Schwendicke F, Tassery H, Ferreira Zandona A, Zero DT, Zimmer S, Doméjean S. How to Intervene in the Caries Process in Children: A Joint ORCA and EFCD Expert Delphi Consensus

Statement. Caries Res. 2020;54(4):297-305. PMID: 32610317 DOI: 10.1159/000507692

3. Riley JL[3rd], Gordan VV, Ajmo CT, Bockman H, Jackson MB, Gilbert GH; Dental PBRN Collaborative Group. Dentists' use of caries risk assessment and individualized caries prevention for their adult patients: findings from The Dental Practice-Based Research Network. Community Dent Oral Epidemiol. 2011;39(6):564-73. PMID: 21726268 PMCID: PMC3193859 DOI: 10.1111/j.1600-0528.2011.00626.x
4. Cagetti MG, Bontà G, Cocco F, Lingstrom P, Strohmenger L, Campus G. Are standardized caries risk assessment models effective in assessing actual caries status and future caries increment? A systematic review. BMC Oral Health. 2018;18(1):123. BMC Oral Health. 2018;18(1):123.
5. Trottini M, Campus G, Corridore D, Cocco F, Cagetti MG, Vigo MI, Polimeni A, Bossù M. Assessing the Predictive Performance of Probabilistic Caries Risk Assessment Models: The Importance of Calibration. Caries Res. 2020;54(3):258-65. PMID: 32516777 DOI: 10.1159/000507276.
6. Zukanovic A. Caries risk assessment models in caries prediction. Acta Med Acad. 2013;42:198-208. PMID: 24308399 DOI: 10.5644/ama2006-124.87.
7. Tellez M, Gomez J, Pretty I, Ellwood R, Ismail AI. Evidence on existing caries risk assessment systems: are they predictive of future caries? Community Dent Oral Epidemiol. 2013;41(1):67–78. PMID: 22978796 DOI: 10.1111/cdoe.12003.
8. Petersson GH, Twetman S, Bratthall D. Evaluation of a computer program for caries risk assessment in schoolchildren. Caries Res. 2002;36(5):327-40. PMID: 12399693 DOI: 10.1159/000065963.

9. Twetman S, Petersson GH, Bratthall D. Caries risk assessment as a predictor of metabolic control in young Type 1 diabetics. Diabet Med. 2005;22(3):312-5. PMID: 15717880 DOI: 10.1111/j.1464-5491.2005.01419.x
10. Fadel H, Al Hamdan K, Rhbeini Y, Heijl L, Birkhed D. Root caries and risk profiles using the Cariogram in different periodontal disease severity groups. Acta Odontol Scand. 2011;69(2):118-24. PMID: 21142897 DOI: 10.3109/00016357.2010.538718.
11. Campus G, Cagetti MG, Sale S, Carta G, Lingström P. Cariogram validity inschoolchildren: a two-year follow-up study. Caries Res. 2012;46(1):16–22. PMID: 22222621 DOI: 10.1159/000334932.
12. Chang J, Lee JH, Son HH, Kim HY. Caries risk profile of Korean dental patientswith severe intellectual disabilities. Spec Care Dentist. 2014;34:201–7. PMID: 25039380 DOI: 10.1111/scd.12047.
13. Sundell AL, Ullbro C, Marcusson A, Twetman S. Comparing caries riskprofiles between 5- and 10- year-old children with cleft lip and/or palateand non-cleft controls. BMC Oral Health. 2015;15:85. PMID: 26208495 PMCID: PMC4514989 DOI: 10.1186/s12903-015-0067-x
14. Campus G, Cagetti MG, Sacco G, Benedetti G, Strohmenger L, Lingström P. Caries risk profiles in Sardinian schoolchildren using Cariogram. Acta Odontol Scand. 2009;67(3):146–52. PMID: 19253062 DOI: 10.1080/00016350902740498.
15. Carta G, Cagetti MG, Cocco F, Sale S, Lingström P, Campus G. Caries-risk profiles in Italian adults using computer caries assessment system and ICDAS. Braz Oral Res. 2015;29(1):S1806-83242015000100306. doi: 10.1590/1807-3107BOR.

16. Pitts NB. Modern concepts of caries measurement. J Dent Res 2004;83:C43–7. doi: 10.1177/154405910408301s09.
17. Landis JR, Koch GG. The measurement of observer agreement for categorical data. Biometrics 1977;33:159-174.
18. Jamieson LM, Thomson WM, McGee R. An assessment of the validity and reliability of dental self-report items used in a National Child Nutrition Survey. Community Dent Oral Epidemiol 2004;32:49-54. doi: 10.1111/j.1600-0528.2004.00126.x.
19. Castiglia P, Campus G, Solinas G, Maida C, Strohmenger L. Children's oral health in Italy: training and clinical calibration of examiners for the National Pathfinder about caries disease. Oral Health Prev Dent. 2007;5(4):255-61.
20. Giacaman RA, Miranda Reyes P, Bravo León V. Caries risk assessment in Chilean adolescents and adults and its association with caries experience. Braz Oral Res. 2013;27(1):7-13. doi: 10.1590/s1806-83242013000100002.
21. Bratthall D, Hänsel Petersson G, Stjernswärd JR. Cariogram Manual. Malmo 2004 available at https://mau.app.box.com/s/pzbw506n8ll7gftp9l2yjb1nhpftzask.
22. Bratthall D, Petersson GH. Cariogram: a multifactorial risk assessment model for a multifactorial disease. Community Dent Oral Epidemiol. 2005;33(4):256-64. doi: 10.1111/j.1600-0528.2005.00233.x.
23. Halasa-Rappel YA, Ng MW, Gaumer G, Banks DA. How useful are current caries risk assessment tools in informing the oral health care decision-making process? J Am Dent Assoc. 2019;150(2):91-102. doi: 10.1016/j.adaj.2018.11.011.

24. Table 1. Cariogram models and cutt-off values considered.

Cariogram	Risk levels (n)	Cut-offs (% of chances to avoid caries) and risk categories				
		Low	Moderate	High		
**Model 1**[a]	3	61-100%	41-60 %	0-40 %		
**Model 2**[b]	3	81-100 %	21-80 %	0-20 %		
		Low	Low/Moderate	Moderate/High	High	
**Model 3**[c]	4	61-100 %	41-60 %	21-40 %	0-20 %	
**Model 4**[d]	4	76-100 %	51-75 %	26-50 %	0-25 %	
		Very low	Low	Moderate	High	Very high
**Model 5**[e]	5	81-100 %	61-80 %	41-60 %	21-40 %	0-20 %

[a]Petsi G, Gizani S, Twetman S, Kavvadia K. Cariogram caries risk profiles in adolescent orthodontic patients with and without some salivary variables . Angle Orthod. 2014 Sep;84(5):891-5; Al Mulla AH, Kharsa SA, Kjellberg H, Birkhed D. Caries risk profiles in orthodontic patients at follow-up using Cariogram. Angle Orthod. 2009 Mar;79(2):323-30. [b]Zukanovic A. Caries risk assessment models in caries prediction. Acta Med Acad. 2013;42:198-208. [c]Chang J, Kim HY. Does caries risk assessment predict the incidence of caries for special needs patients requiring general anesthesia? Acta Odontol Scand. 2014;72:721-8; Petersson GH, Fure S, Bratthall D. Evaluation of a computer-based caries risk assessment program in an elderly group of individuals. Acta Odontol Scand. 2003;61:164-71. [d]Twetman S, Petersson GH, Bratthall D. Caries risk assessment as a predictor of metabolic control in young Type 1 diabetics. Diabet Med. 2005;22:312-5. [e]Campus G, Cagetti MG, Sale S, Carta G, Lingström P. Cariogram validity in schoolchildren: a two-year follow-up study. Caries Res. 2012;46:16-22; Petersson GH, Twetman S. Caries risk assessment in young adults: a 3 year validation of the Cariogram model. BMC oral health. 2015;15:17.

Table 2. Description of the sample.

Samples	C_{young} *n=957*	C_{old} *n=862*	A_{young} *n=86*	A_{old} *n= 480*
Gender	Males n=473 (49.37)<br>Females n=485 (50.63)	Males n= 421 (48.84%)<br>Females n=441 (51.16%)	Males n=39 (45.35%)<br>Females n=47 (54.65%)	Males n=254 (52.92%)<br>Females n=226 (47.08%)
Age	6.49±0.71 years (range 7-9)	8.16±0.94 years (range 9-11)	23.37±3.11 years (range 21-27)	40.72±9.41 years (range 30-45)
Caries-free	409 (47.91%)	368 (42.69%)	26 (30.23%)	(36.46%)
Caries Experience	2.37±4.05 (range 0-19)	3.22±4.49 (range 0-23)	2.91±3.30 (0-13)	3.25±4.15 (0-21)
D/d	1.38±2.62 (range 0-17)	2.51±3.78 (range 0-22)	0.26±0.64 (range 0-3)	0.24±0.72 (0-8)
M/m	---	---	0.09±0.62 (range 0-5)	0.49±1.54 (0-9)
F/f	0.4±1.74 (range 0-7)	0.63±2.21 (range 0-10)	2.56±3.00 (range 0-11)	2.51±4.56 (0-20)

Table 3. Caries risk in the four samples using the five Cariogram models.

	Model 1 (3 Risk categories)			
	C_{young}	C_{old}	A_{young}	A_{old}
Low	487 (50.94)	449 (52.09)	55 (63.95)	12 (2.50)
Moderate	195 (20.40)	165 (19.14)	20 (23.26)	386 (80.42)
High	274 (28.66)	248 (28.77)	32 (37.21)	82 (17.08)
	Model 2 (3 Risk categories)			
Low	332 (34.73)	309 (35.85)	32 (37.21)	--
Moderate	432 (45.19)	378 (43.85)	52 (60.47)	479 (99.79)
High	192 (20.08)	175 (20.30)	2 (2.33)	1 (0.21)
Cohen Kappa	*K=0.63 SE=0.03 p<0.01*	*K=0.63 SE=0.02 p<0.01*	*K=0.40 SE=0.07 p<0.01*	*K=0.71 SE=0.05 p=0.02*

	Model 3 (4 Risk categories)			
	C_{young}	C_{old}	A_{young}	A_{old}
Low	487 (50.94)	449 (52.09)	55 (63.95)	12 (2.50)
Low/Moderate	195 (20.40)	165 (19.14)	20 (23.26)	386 (80.42)
Moderate/High	82 (8.58)	73 (8.47)	9 (10.47)	81 (16.88)
High	192 (20.08)	175 (20.30)	2 (2.33)	1 (0.21)
	Model 4 (4 Risk categories)			
Low	332 (34.73)	309 (35.85)	37 (43.02)	--
Low/Moderate	249 (26.05)	213 (24.71)	29 (33.72)	152 (31.67)
Moderate/High	183 (19.14)	165 (19.14)	15 (17.44)	326 (67.92)
High	192 (20.08)	175 (20.30)	5 (5.81)	2 (0.42)
Cohen Kappa	*K=0.62 SE=0.02 p<0.01*	*K=0.62 SE=0.02 p<0.01*	*K=0.44 SE=0.07 p<0.01*	*K=0.14 SE=0.03 p<0.01*

	Model 5 (5 Risk categories)			
	C_{young}	C_{old}	A_{young}	A_{old}
Very Low	332 (34.73)	309 (35.85)	55 (63.95)	--
Low	155 (16.21)	140 (16.24)	20 (23.26)	12 (2.50)
Moderate	195 (20.40)	165 (19.14)	--	386 (80.42)
High	82 (8.58)	73 (8.47)	9 (10.47)	81 (16.88)
Very High	192 (20.08)	175 (20.30)	2 (2.33)	1 (0.21)

Table 4. Multinomial regression analysis. Cariogram risk categories as dependent variables .

			C_{young} Group		
Risk variables	Model 1	Model 2	Model 3	Model 4	Model 5
	OR (SE) $_{95\%}$CI	*OR (SE) $_{95\%}$CI*	*OR (SE) $_{95\%}$CI*	*OR (SE) $_{95\%}$CI*	*OR (SE) $_{95\%}$CI*
Caries experience	1.51(0.06) 1.40 / 1.63	1.53 (0.07) 1.39 / 1.67	1.40 (0.05) 1.31 / 1.49	1.72 (0.07) 1.58 / 1.87	1.59 (0.06) 1.49 / 1.70
Related disease	--	--	--	--	--
Diet Contents	7.49 (5.03) 2.01 / 27.93	22.09 (14.27) 6.23 / 78.34	7.55 (5.10) 2.01 / 28.36	9.27 (3.91) 4.05 / 21.18	2.48 (0.82) 1.30 / 4.74
Diet Frequency	2.85 (0.38) 2.20 / 3.70	2.73 (0.46) 1.96 /3.80	3.17 (0.43) 2.43 / 4.13	2.99 (0.40) 2.29 / 3.87	2.67 (0.31) 2.12 / 3.36
Plaque Amounts	2.24 (0.44) 1.53 / 3.28	299.53 (158.39) 106.26 / 844.38	6.13 (1.14) 4.26 / 8.83	127.92 (38.26) 71.17 / 229.90	11.71(2.00) 8.38 / 16.37
Mutans Streptococci	2.32 (0.15) 2.05 / 2.62	2.71 (0.24) 2.28 / 3.22	2.32 (0.13) 2.07 / 2.61	3.02 (0.23) 2.60 / 3.50	2.43 (0.141) 2.18 / 2.73
Fluoride Program	1.78 (0.09) 1.58 / 2.06	1.84 (0.08) 1.61 / 2.24	1.48 (0.04) 1.15 / 2.02	1.25 (0.05) 0.94 / 1.54	1.25 (0.05) 0.94 / 1.54
			C_{old} Group		
Risk variables	Model 1	Model 2	Model 3	Model 4	Model 5
	OR (SE) $_{95\%}$CI	*OR (SE) $_{95\%}$CI*	*OR (SE) ($_{95\%}$CI)*	*OR (SE) $_{95\%}$CI*	*OR (SE) $_{95\%}$CI*
Caries experience	1.69 (0.09) 1.52 / 1.87	1.50 (0.08) 1.36 / 1.66	1.43 (0.05) 1.33 / 1.54	1.81 1.64 / 2.00	1.63 (0.06) 1.51 / 1.76
Related disease	--	--	--	--	--
Diet Contents	3.25 (0.48) 2.43 / 4.35	3.27 (0.61) 2.27 / 4.70	3.84 (0.58) 2.85 / 5.17	3.23 (0.46) 2.43 / 4.28	3.20 (0.41) 2.49 / 4.12
Diet Frequency	16.06 (17.33) 1.94 / 13.15	9.02 (6.00) 2.45 / 33.21	11.40 (12.23) 1.39 / 93.33	4.54 (2.02) 1.90 / 10.86	1.40 (0.49) 0.70 / 2.80
Plaque Amounts	1.55 (0.33) 1.01 / 2.36	405.56 (214.73) 143.68 / 1144.80	5.51 (1.11) 3.71 / 8.17	127.19 (39.15) 69.58 / 232.50	12.27 (2.24) 8.57 / 17.56
Mutans Streptococci	2.42 (0.17) 2.11 / 2.79	2.60 (0.24) 2.18 / 3.12	2.27 (0.14) 2.0 / 2.56	3.00 (0.24) 2.57 / 3.50	2.39 (0.14) 2.12 / 2.67
Fluoride Program	2.03 (0.12) 1.71 / 2.82	1.43 (0.03) 0.95 / 1.95	1.73 (0.04) 0.88 / 2.70	2.01 (0.05) 1.09 / 3.10	2.03 (0.05) 1.11 / 3.26
			A_{young} Group		
Risk variables	Model 1	Model 2	Model 3	Model 4	Model 5
	OR (SE) $_{95\%}$CI	*OR (SE) P-value $_{95\%}$CI*	*OR (SE) P-value ($_{95\%}$CI)*	*OR (SE) $_{95\%}$CI*	*OR (SE) $_{95\%}$CI*
Caries experience	17.60 (20.74) 3.17 / 34.66	5.83 (2.85) 2.23 / 15.18	32.49 (34.72) 4.00 / 263.85	2.66 (0.719) 1.56 / 4.52	10.45 (4.68) 4.35 / 25.13
Related disease	--	--	--	--	--
Diet Contents	38.66 (55.28) 2.35 / 637.47	4.36 (2.33) 0.53 / 12.45	16.911 (16.30) 2.69 / 111.90	5.83 (2.26) 2.73 / 12.45	8.24 (3.86) 3.29 / 20.64
Diet Frequency	35.13 (45.94) 2.71 / 455.92	3.66 (1.72) 1.45 / 9.20	8.44 (5.65) 2.27 / 31.34	3.32 (1.08) 1.76 / 6.27	5.18 (1.96) 2.46 / 10.88
Plaque Amounts	55.69 (106.22) 1.32 / 339.80	1.81 (1.77) 0.26 / 12.37	22.90 (34.84) 1.162 / 451.47	2.70 (2.02) 0.63 / 11.68	3.35 (2.56) 0.74 / 15.04
Mutans Streptococci	238.38 (429.67) 6.97 / 815.87	4.57 (2.30) 1.71 / 12.25	81.96 (100.84) 7.35 / 913.92	4.47 (1.70) 2.12 / 9.44	10.38 (5.17) 3.91 / 27.56
Fluoride Program	126.60 (41.07) 11.07 / 3388.01	23.45 (21.48) 3.89 / 141.14	97.64 (182.57) 16.56 / 553.66	11.15 (5.77) 4.04 / 30.76	85.99 (71.36) 16.90 / 437.35
Saliva Secretion	75.71 (22.75) 3.15 / 181.69	4.17 (0.91) 1.01 / 16.40	11.78 (1.37) 2.10 / 26.13	3.12 (1.40) 1.30 / 7.52	7.39 (3.85) 2.67 / 20.44
Buffer Capacity	36.08 (60.46) 1.35 / 96.29	3.94 (1.60) 0.73 / 21.03	5.55 (1.29) 0.87 / 35.77	5.84 (2.76) 1.67 / 20.46	8.07 (5.50) 2.12 / 30.74
			A_{old} Group		
Risk variables	Model 1	Model 2	Model 3	Model 4	Model 5
	OR (SE) $_{95\%}$CI	*OR (SE) $_{95\%}$CI*	*OR (SE) $_{95\%}$CI*	*OR (SE) $_{95\%}$CI*	*OR (SE) $_{95\%}$CI*
Caries experience	3.61 (2.06) 1.18 / 11.03	convergence not achieved	2.51 (0.92) 1.22 / 5.14	convergence not achieved	2.51 (0.92) 1.22 / 5.14
Related disease	omitted		omitted		omitted
Diet Contents	5.89 (2.54) 2.52 / 13.72		5.81 (2.32) 2.5 / 12.73		5.81 (2.32) 2.5 / 12.73
Diet Frequency	4.16 (1.54) 1.36 / 10.22		3.76 (1.82) 1.44 / 9.72		3.76 (1.82) 1.44 / 9.72
Plaque Amounts	3.00 1.60) 1.52 / 10.12		2.34 (0.62) 0.88 / 4.05		1.51 (0.84) 0,90 / 2.13
Mutans Streptococci	6.81 (3.56) 2.44 / 18.94		5.29 (2.00) 2.52 / 11.13		5.29 (2.00) 2.52 / 11.13
Fluoride Program	3.16 (1.26) 1.45 / 6.91		0.77 (0.86) 0.08 / 6.82		0.77 (0.86) 0.08 / 6.82
Saliva Secretion	3.46 (0.64) 0.71 / 6.48		2.51 (0.75) 1.00 / 6.08		2.51 (0.75) 1.00 / 6.08
Buffer Capacity	3.82 (1.14) 0.81 / 4.72		7.28 (1.34) 0.90 / 34.62		7.28 (1.34) 0.90 / 34.62

Click here to access/download
Supporting Information
STROBE_checklist_cross-sectional.doc

Click here to access/download

Supporting Information

Cariogram 2009 .pdf

Click here to access/download
Supporting Information
paper2012.pdf

paper2012

Click here to access/download
Supporting Information
paper2015.pdf

Supporting Information row data

Click here to access/download
Supporting Information
row datai.xlsx
